# Comparative study on intestinal microbiome composition and function in young and adult Hainan gibbons (*Nomascus hainanus*)

**DOI:** 10.7717/peerj.13527

**Published:** 2022-06-08

**Authors:** Yimeng Li, Yu Bi, Liangliang Yang, Kun Jin

**Affiliations:** 1 Institute of Forest Ecology-Environment and Nature Conservation, Chinese Academy of Forestry, Beijing, China; 2 Beijing Museum of Natural History, Beijing, China; 3 Hainan Institute of National Park, Haikou, China; 4 Research Institute of Natural Protected Area, Chinese Academy of Forestry, Beijing, China; 5 Key Laboratory of Biodiversity Conservation of National Forestry and Grassland Administration, Beijing, China

**Keywords:** Hainan gibbon (*Nomascus hainanus*), Intestinal microbiome, Metagenome

## Abstract

The Hainan gibbon is one of the most endangered primates in the world, with a small population size, narrow distribution range, and high inbreeding risk, which retains the risk of species extinction. To explore the composition and functional differences of the intestinal microbiome of Hainan gibbons at different ages, the faecal microbiomes of young and adult Hainan gibbons were analysed using metagenome sequencing. The results showed that the dominant phyla in the intestinal tract of young and adult Hainan gibbons were Firmicutes and Bacteroidetes, and the dominant genus was *Prevotella*. Linear discriminant analysis effect size analysis showed that Firmicutes, *Ruminococcus*, *Clostridium*, and *Butyrivibrio* were significantly more abundant in adults than in young, whereas Bacteroidetes, Proteobacteria, *Prevotella*, and *Bacteroides* were significantly more abundant in young than in adults. In terms of gene function, the adult Hainan gibbon intestinal microbiome generally harboured a higher abundance of genes related to metabolic processes, such as carbohydrate, amino acid, and nucleotide metabolism. This may be due to adaptive advantages for adult Hainan gibbons, such as stable and mature intestinal microbiome composition, which allows them to utilise diverse foods efficiently. In summary, this study helps understand the dynamic changes in the intestinal microbiome of young and adult Hainan gibbons and plays a key role in the health monitoring and rejuvenation of their population.

## Introduction

The Hainan gibbon (*Nomascus hainanus*) is a species endemic to China, belonging to the Primates order, Hylobatidae family, and *Nomascus* genus. Historically, Hainan gibbons have been widely distributed in tropical rain forests on Hainan Island. Since the 1950s, due to indiscriminate poaching and a reduction in natural habitat areas, the population of Hainan gibbons has decreased rapidly. At present, they are only distributed in the Hainan Tropical Rain Forest National Park, with a total of 35 individuals in five family groups (Zhang, 2021), making it one of the least prevalent primates in the world. The Hainan gibbon is listed as a Class I key protected wild species in China, and the International Union for Conservation of Nature has assessed the status of this species as ‘critically endangered’ (Geissmann & Bleisch, 2020). Since the 1970s, researchers have carried out a series of studies on Hainan gibbons, focusing on habitat range (Liu & Tan, 1990; Tang & Jin, 2021), population size (Fellowes et al., 2008), behaviour (Zhou, Chen & Wei, 2008), genetic diversity (Li, Wei & Zhou, 2010; Han, 2019), and feeding habits (Tang, Bi & Jin, 2021). However, little research has been conducted on the intestinal microbiome of Hainan gibbons.

The bacteria in the host intestine are variable, and their composition is affected by host genotype (Kovacs et al., 2011), diet (Li et al., 2017; Singh et al., 2017), age (Odamaki et al., 2016), habitat (Dulski, Kozowski & Ciesielski, 2020), disease (Owyang & Wu, 2014), and other factors. A large number of studies have confirmed that the dynamic balance of the intestinal microbiome is closely related to host immunity, development, nutrient absorption, and energy metabolism (Bäckhed et al., 2004; Round & Mazmanians, 2009; Myer et al., 2017), and its potential significance in maintaining host health is becoming increasingly prominent. Therefore, studies on the intestinal microbiome have attracted increasing attention in the field of endangered species protection. Non-human primates have closely related to humans; thus, the study of intestinal microbiome in non-human primates is of significance. To date, intestinal microbiome composition of a variety of non-human primates, including lemurs and lorises (McKenney, Rodrigo & Yoder, 2015), New World monkeys and Old World monkeys (Amato et al., 2013; Sun et al., 2016), and apes (Ochman et al., 2010) has been reported. In general, the intestinal microbiome of non-human primates is composed of bacteria from 12 phyla such as Bacteroidetes, Firmicutes, Proteobacteria, Tenericutes, Actinobacteria.

In this study, we examined the intestinal microbiome of young and adult Hainan gibbons in the dry season in order to explore and compare the composition and functional differences in the intestinal microorganisms of Hainan gibbons at different ages, in order to provide a theoretical basis for the protection of this extremely endangered species.

## Materials and Methods

### Ethics statement

This study was carried out in accordance with the recommendations of the Institute of Animal Care and the Ethics Committee of Chinese Academy of Forestry (IACUC number: BJ2021-006). The Ethics Committee of Chinese Academy of Forestry also approved the protocol. The management authority of Hainan Tropical Rain Forest National Park approved the collection of Hainan gibbon fecal samples (Field permit number: 2021-315).

### Study areas

Faecal samples were collected in Hainan Tropical Rainforest National Park (18°57′–19°11′N, 109°3′–109°17′E), which is located in the mountainous area southwest of Hainan Island, in Changjiang County and Baisha County. The total area of the National Park is approximately 4,269 km^2^, and the altitude is approximately 590–1,560 m. The dry season is from November to April in the following year, and the rainy season is from May to October. The average annual precipitation is 1,657 mm, and the average annual temperature is 21.3 °C. The Hainan gibbon mainly feeds on succulent mature fruits and tender leaves of plants such as *Artocarpus styracifolius*, *Diospyros maclurei*, *Apodytes dimidiata*, *Endospermum chinensis*, and *Garcinia oblongifolia* (Tang, Bi & Jin, 2021). When food is relatively scarce in the dry season, these plant species are relatively more abundant than in the rainy season. During our sampling period, we observed the following: young Hainan gibbons prefer to eat fruits compared with the adults, the excreted pits can be observed in their collected faeces, and adults eat more tender leaves than young Hainan gibbons.

### Sample collection

A total of 11 fresh faecal samples were collected from January to May 2021, including samples from six young males (Y1–Y6, approximately 7 years old) and five adult males (A1–A5, approximately 20 years old) from five family groups. The sampling procedure was as follows: experienced members of the Hainan gibbons monitoring team of the Bawangling Forestry Bureau assisted in the collection of faecal samples. We arrived at the monitoring point before the chirp of the Hainan gibbon in the morning. After hearing Hainan gibbon chirp, we found the family group of Hainan gibbons according to the chirp and tracked it with a telescope. Individuals were identified based on the individual size, morning chirp, and other behaviours, and faeces were collected immediately after finding the Hainan gibbon’s defaecation. To ensure that the faeces were fresh, only samples from individuals with observed defaecation on site were collected. We wore disposable sterile gloves to collect the faeces. The dirt-stained part was removed, and the central part was placed into a 15 ml sterile centrifuge tube, sealed, labelled, and retained in a mobile refrigerator until taken to the laboratory for final storage at −80 °C.

### DNA extraction and sequencing

A DNeasy PowerSoil Pro Kit (Qiagen, Hilden, Germany) was used to extract faecal DNA from the adult and young Hainan gibbons. Detailed extraction steps were performed according to the manufacturer’s instructions. The DNA concentration was determined by Qubit dsDNA HS Assay Kit (Life Technologies, Carlsbad, CA, USA). DNA was fragmented into an average size of approximately 400 bp with Covaris M220 instrument (Gene Company Ltd., Hong Kong, China) for paired-end library construction, and then the paired-end library was constructed using NEXTFLEX Rapid DNA-Seq (Bioo Scientific, Austin, TX, USA). Paired-end sequencing was done with the Illumina NovaSeq 6000 platform (Illumina Inc., San Diego, CA, USA) at Biomarker Bioinformatics Technology Co., Ltd. (Beijing, China) using the NovaSeq 6000 S4 Reagent Kit following the manufacturer’s instructions.

### Bioinformatic analysis

Representative sequences of a non-redundant gene catalogue were aligned to those in the NCBI NR database with an e-value cut-off of 1e−5 using Diamond (version 0.8.35) for taxonomic annotations. Cluster of orthologous groups of proteins (COG) annotation for the representative sequences was performed using Diamond (version 0.8.35) against eggNOG database with an e-value cut-off of 1e−5. The KEGG annotation was conducted using Diamond (version 0.8.35) against the Kyoto Encyclopedia of Genes and Genomes database with an e-value cut-off of 1e−5. Carbohydrate-active enzymes annotation was conducted using hmmscan against the CAZy database with an e-value cut-off of 1e−5. The relative abundance data of functional categories are shown as mean ± SD. The normality of data was tested using the Kolmogorov–Smirnov test. For statistical analysis, t-test for independent samples was performed to compare data between two groups. Linear discriminant analysis effect size (LEfSe) was used to determine differentially distributed bacterial taxa in young and adult Hainan gibbons. The LEfSe analysis was performed online using the Galaxy workflow framework (http://huttenhower.sph.harvard.edu/galaxy/). The raw data obtained in this study have been submitted to the NCBI Sequence Read Archive (accession number SRR18250961).

## Results

### Statistics of metagenome sequencing data

The sequencing data statistics are shown in Table S1. A total of 69,776.55 Mbp raw data were obtained from 11 Hainan gibbon faeces samples. After quality control, a total of 59,074.57 Mbp of vaild data were obtained, corresponding to 198,717,889 reads. The percentage of bases with quality values ≥20 or ≥30 reached more than 97% and 93%, respectively, indicating that the sequencing data showed high reliability.

### Intestinal microbiome composition of young and adult Hainan gibbon

**Figure 1:**
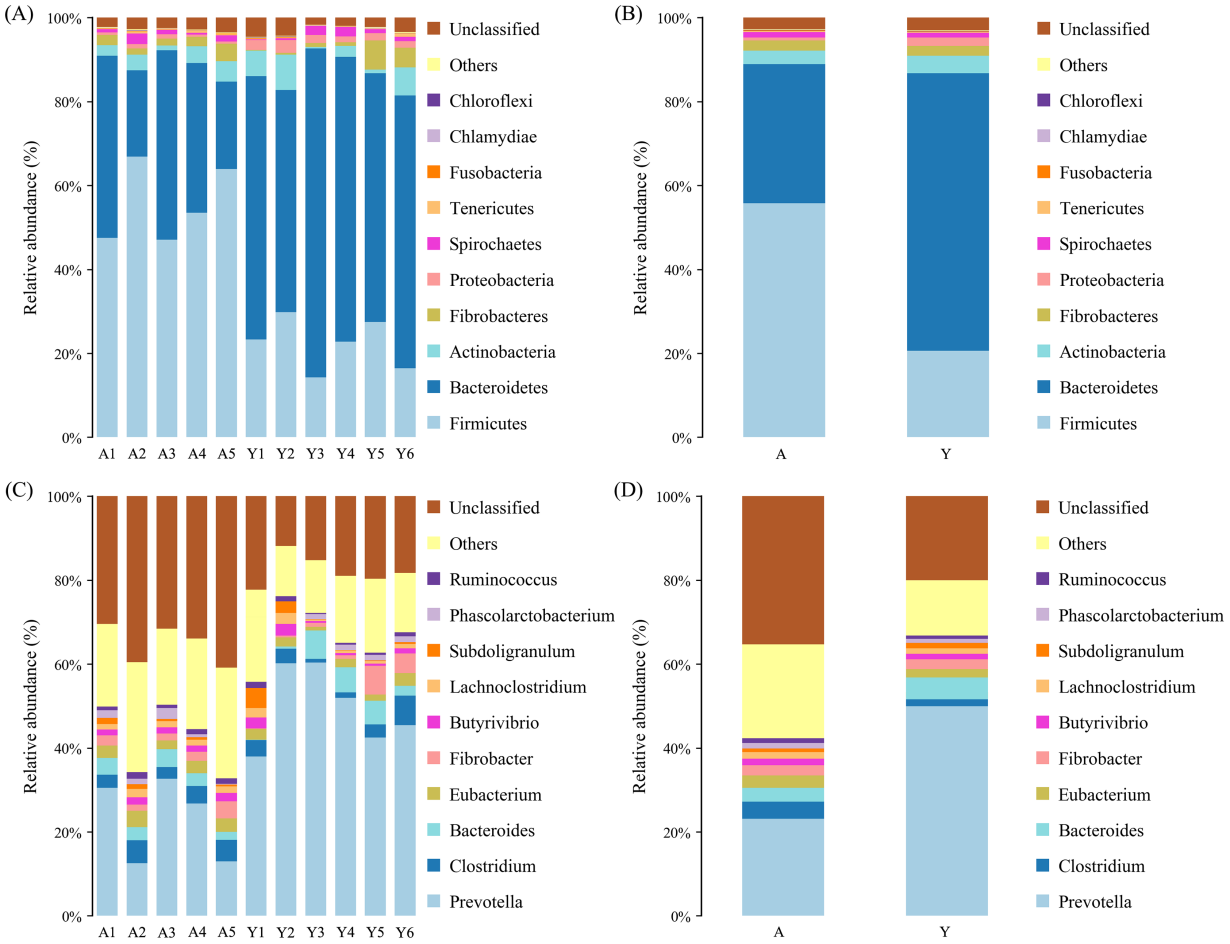
Histogram of relative abundance at phylum and genus levels. Relative abundance (%) of the 10 most abundant bacteria phyla ((A) for Individuals, (B) for groups) and genera ((C) for individuals, (D) for groups) obtained from 11 fecal samples of Hainan gibbons. Others: Bacteria taxa with ≤1% abundance; Unclassified: Sequences which could not be classified.

At the phylum level (Figs. 1A and 1B), Firmicutes (Y: 0.21 ± 0.07%; A: 0.56 ± 0.09%), and Bacteroidetes (Y: 0.69 ± 0.10%; A: 0.33 ± 0.12%) were the main dominant phyla in young and adult Hainan gibbons, followed by Actinobacteria (Y: 0.01 ± 0.01%; A: 0.05 ± 0.03%), Fibrobacteres (Y: 0.03 ± 0.03%; A: 0.02 ± 0.01%), and Spirochaetes (Y: 0.02 ± 0.01%; A: 0.01 ± 0.01%). At the genus level (Figs. 1C and 1D), *Prevotella* (Y: 0.52 ± 0.09%; A: 0.23 ± 0.10%) was the main dominant genera, followed by *Bacteroides* (Y: 0.06 ± 0.01%; A: 0.03 ± 0.01%), *Clostridium* (Y: 0.02 ± 0.01%; A: 0.04 ± 0.01%), and *Eubacterium* (Y: 0.01 ± 0.01%; A: 0.03 ± 0.01%).

### Lefse analysis

**Figure 2:**
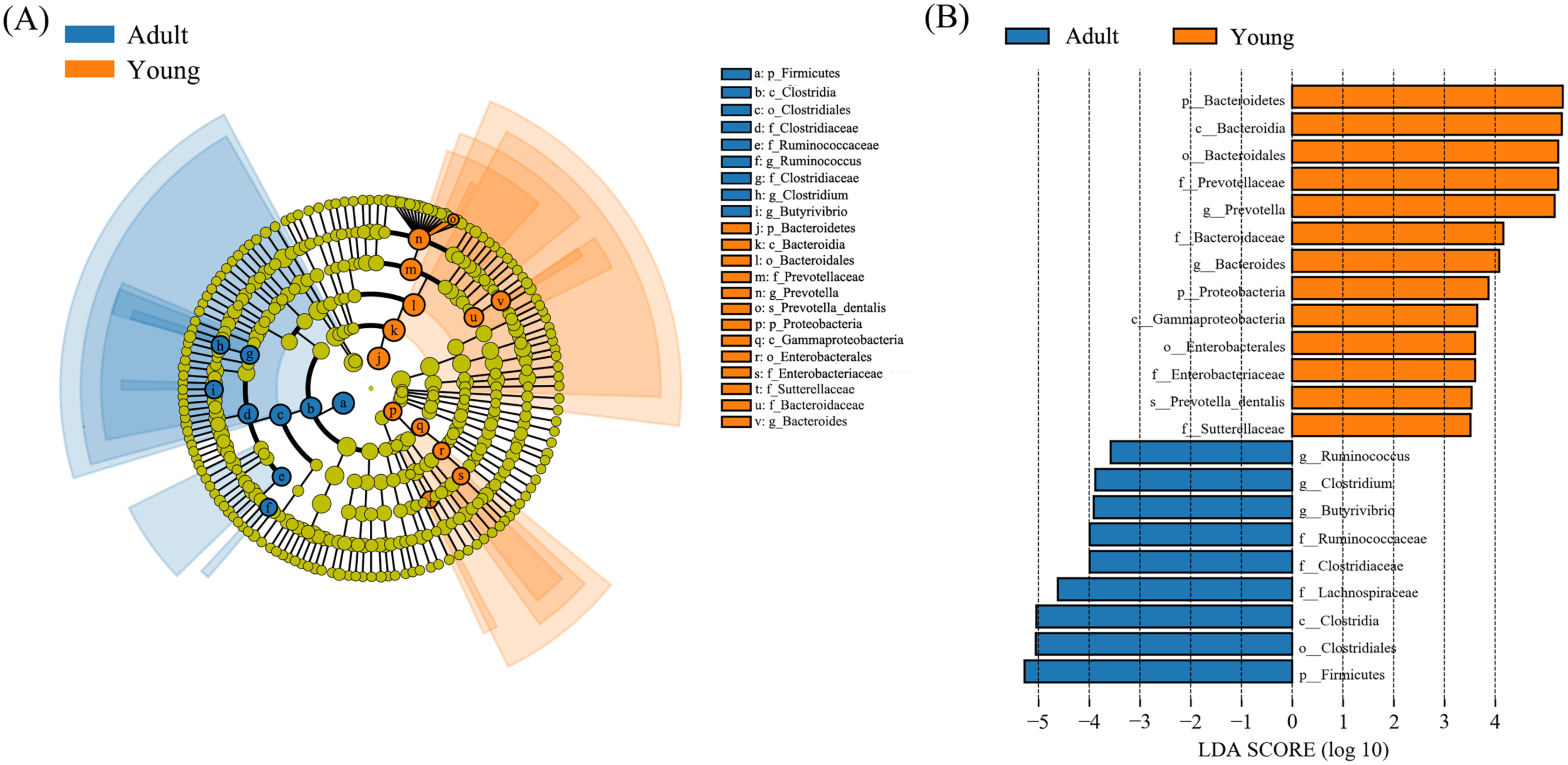
LefSe analysis. (A) Cladogram based on LefSe analysis showing the taxa with significant differences between adult and young Hainan gibbons. Taxonomic hierarchies were arranged from the inside (lower taxonomic level) to the outside (higher taxonomic level). Orange and blue nodes in the phylogenetic tree represent differentially abundant taxa in the two groups. Yellow nodes represent taxa with no significant difference. (B) Taxa with significant difference that have an LDA score larger than the threshold value of 3.5; letters in front of taxa represent taxonomic level (p = phylum, c = class, o = order, f = family, g = genus, s = species).

LEfSe analysis identified 22 taxa that showed significantly different abundances between young and adult Hainan gibbons (Fig. 2). At the phylum level, the relative abundance of Firmicutes was significantly higher in adults than in young, whereas Bacteroidetes and Proteobacteria were significantly higher in young than in adults. At the genus level, *Ruminococcus*, *Clostridium*, and *Butyrivibrio* were significantly higher in adults than in young, whereas *Prevotella* and *Bacteroides* were significantly higher in young than in adults.

### Analysis of functional gene difference of intestinal microbiome in young and adult Hainan gibbon

**Figure 3:**
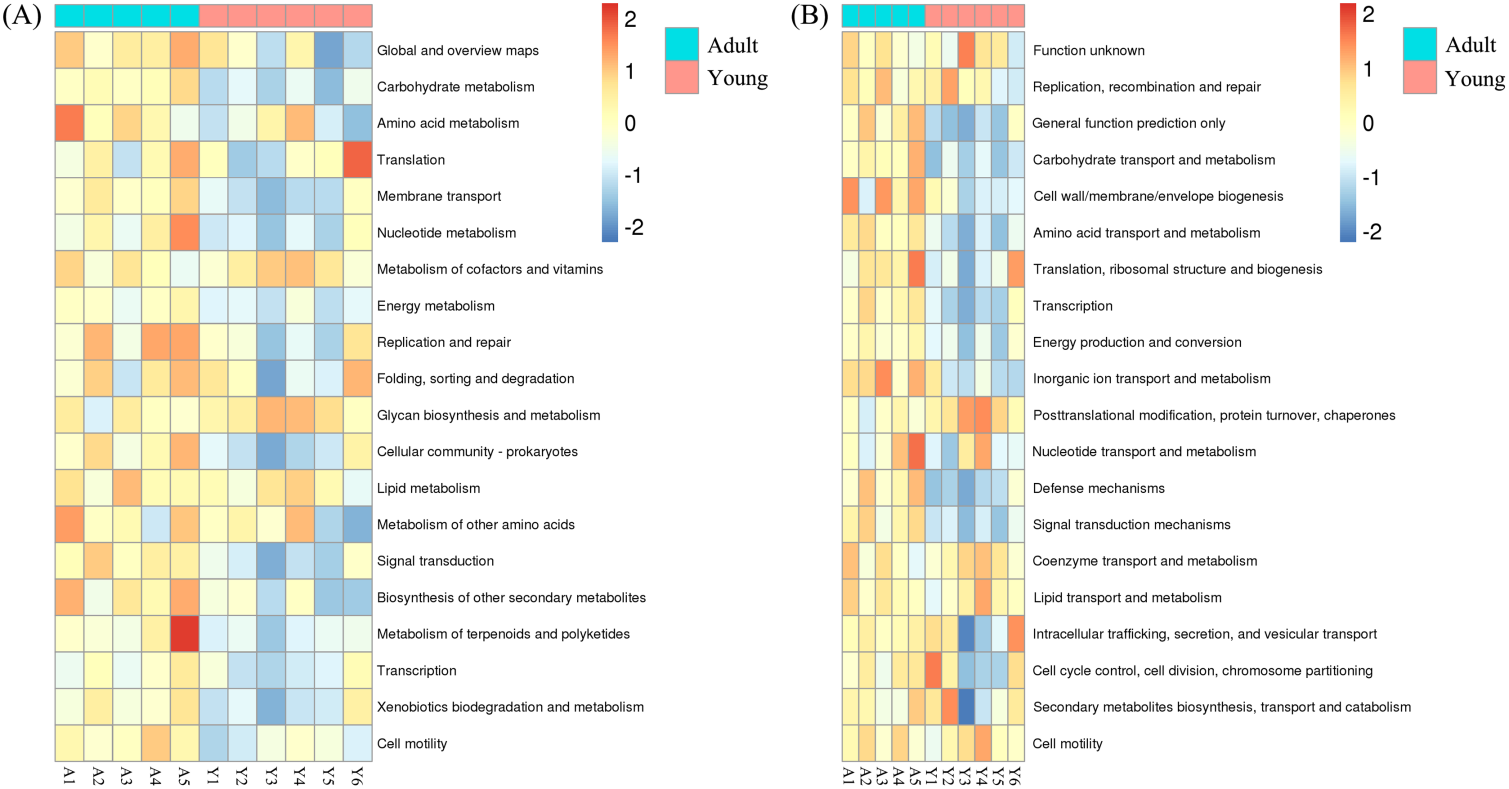
Function heatmap. The heatmaps show top 20 relative abundance of (A) KEGG pathways (level 2 function) and (B) COG categories (class function) for the microbial metagenome of the adult and young Hainan gibbons.

**Figure 4:**
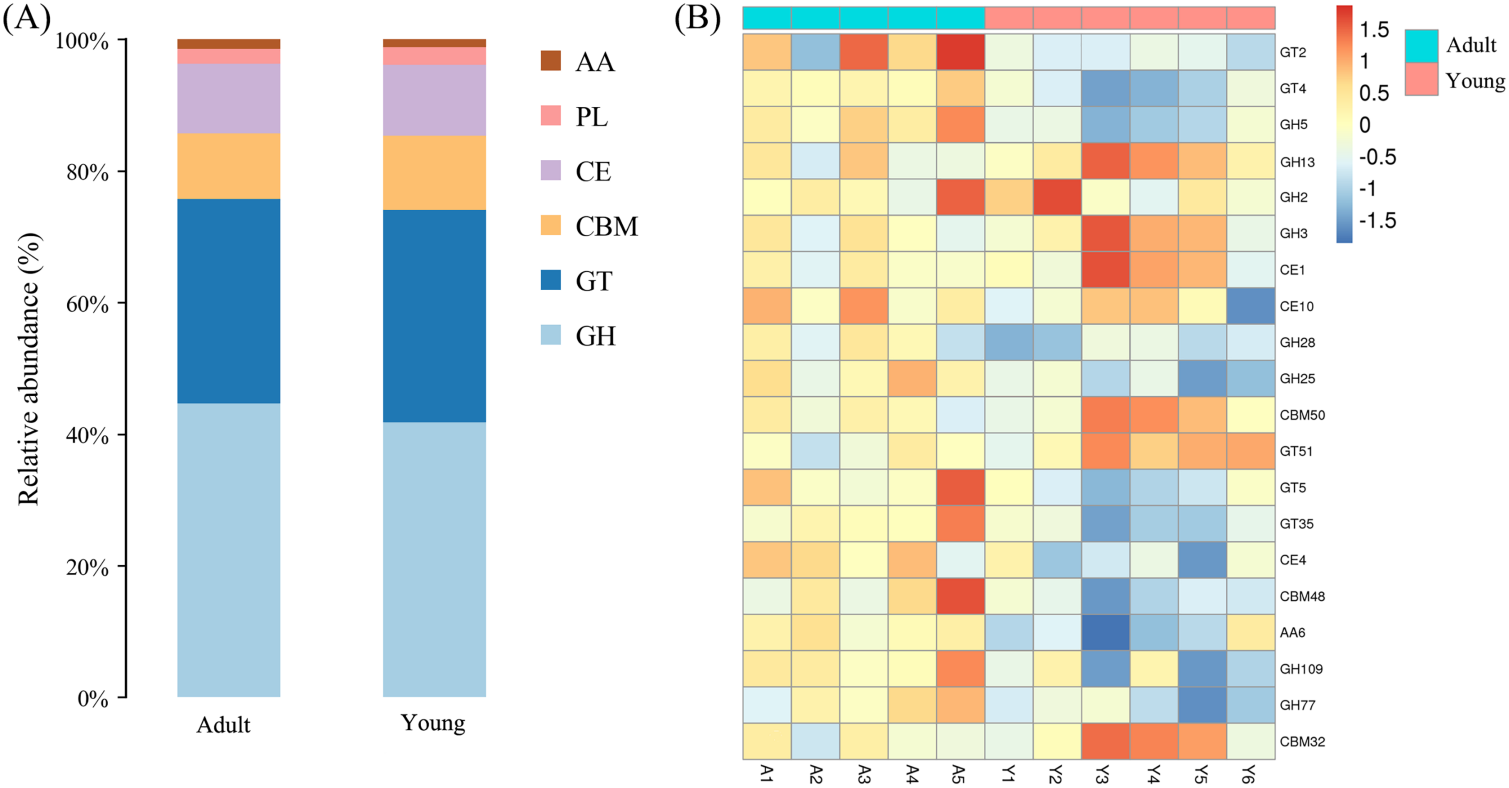
CAZy analysis. (A) Functional classification histogram of adult and young Hainan gibbons. (B) The heatmaps show top 20 relative abundance of CAZymes for the microbial metagenome of the adult and young Hainan gibbons.

Heatmaps were constructed to explore the differences in the abundance of genes related to various KEGG, COG (Fig. 3; Tables S2 and S3) and CAZy (Fig. 4) categories between young and adult Hainan gibbons. Based on KEGG annotation for adult Hainan gibbons, the abundance of the carbohydrate metabolism, nucleotide metabolism, membrane transport, energy metabolism, Cellular community—prokaryotes, signal transduction, biosynthesis of other secondary metabolites, metabolism of terpenoids and polyketides and Xenobiotics biodegradation and metabolism categories was significantly higher than in young Hainan gibbons. Based on COG annotation for adult Hainan gibbons, the abundance of the general function prediction only, carbohydrate transport and metabolism, amino acid transport and metabolism, transcription, energy production and conversion, Inorganic ion transport and metabolism, defense mechanisms, and signal transduction mechanisms categories was significantly higher than in young Hainan gibbons. For young Hainan gibbons, the abundance of the posttranslational modification, protein turnover, chaperones category was significantly higher than in adult Hainan gibbons.

Based on CAZy annotation, the database divides carbohydrate active enzymes into the following six classes: auxiliary activities (AAs), glycosyltransferases (GTs), carbohydrate esterases (CEs), polysaccharide lyases (PLs), glycoside hydrolases (GHs), and carbohydrate-binding modules (CBMs). The results showed that the sequences were enriched in 109 GH families, 73 GT families, 72 CBM families, 18 PL families, 15 CE families, and 9 AA families. The content of GHs in adult and young Hainan Gibbons was the highest, accounting for 43.31% and 41.81% of the total carbohydrate active enzymes, respectively, followed by GTs (32.42%, 32.28%), CBMs (9.94%, 11.26%), CEs (10.62%, 10.74%) and PLs (2.27%, 2.70%), whereas, the abundance of AAs was the lowest, accounting for only 1.44% and 1.19% of the total carbohydrate active enzymes, respectively. GHs hydrolyse glycosidic bonds between carbohydrate molecules. GHs involved in the degradation of cellulose, hemicellulose and oligosaccharide were abundant in the intestinal tract of the two groups of Hainan gibbons. Among the 20 most abundant carbohydrate enzymes, GH5 is the predominant enzyme involved in cellulose degradation, GH2 and GH3 play a major role in oligosaccharide degradation, and GH28 plays an important role in hemicellulose degradation. The results showed that the abundance of enzyme involved in cellulose and hemicellulose degradation in the adult Hainan gibbon group was higher than that in the young Hainan gibbon group, whereas, the abundance of enzymes involved in oligosaccharide degradation in the young Hainan gibbon group was higher than that in the adult Hainan gibbon group.

## Discussion

In this study, metagenome sequencing was used to analyse the composition and functional differences in the intestinal microbiome between young and adult Hainan gibbons, with the aim to explore the influence of age-related factors on the host intestinal microbiome.

Firmicutes and Bacteroidetes were the dominant phyla in the intestines of young and adult Hainan gibbons. Previous studies have reported the composition of intestinal microbiome in a variety of non-human primates such as *Macaca thibetana* (Sun et al., 2016), *Alouatta pigra* (Amato et al., 2014), *Gorilla gorilla gorilla* (Pafčo et al., 2019), and *Rhinopithecus bieti* (Xu et al., 2015). The proportion of Bacteroidetes and Firmicutes is the highest in the intestinal microbiome of most non-human primates. In this study, the proportion of Firmicutes increased with age and that of Bacteroidetes decreased with age. This may be because the immune system of young Hainan gibbons is not mature yet and the composition of the intestinal microbiome is not stable. *Bacteroides* can promote immune system development to enhance host immunity (Hooper, 2004) and maintain intestinal microbial ecological balance (Hooper et al., 2001; Sears, 2005); therefore, it is highly abundant in the intestines of young Hainan gibbons. In addition, the intestine of Hainan gibbon contains Fibrobacteres members, which mainly exist in the rumen of ruminants, where cellulase is present in the periplasm; Fibrobacteres members can decompose cellulose and enable hosts to absorb it (Jewell et al., 2013). Zhou et al. (2014) also confirmed that the intestinal microbiome composition of golden monkeys is similar to that of ruminants, such as cattle, and this is related to their similar dietary structure. *Prevotella*, *Bacteroides*, and *Clostridium* were the dominant bacteria at the genus level. These bacteria are generally closely related to host food digestion, and they can help leaf-eating non-human primates degrade structural carbohydrates such as cellulose in leaves (Amato et al., 2014), whereas *Bacteroides* members can help decompose non-structural carbohydrates such as fructose in fruits.

The results of the LefSe analysis showed that in adult Hainan gibbons, *Ruminococcus*, *Clostridium*, and *Butyrivibrio* were significantly more abundant than in young Hainan gibbons, whereas *Prevotella* and *Bacteroides* were significantly more abundant in young gibbons. This result is similar to the result of high abundance of *Prevotella* in the intestine of *Macaca mulatta* (Zhou, 2019). *Prevotella* plays an important role in the decomposition and utilization of hemicellulose, pectin, starch, and monosaccharides (Flint, 2004); this is related to the consumption on fruits, which contain a large amount of pectin. It is apparent that adult Hainan gibbons are better adapted to digesting structural carbohydrates in leaves (Ding et al., 2001), whereas young Hainan gibbons are better adapted to decomposing non-structural carbohydrates in fruits. Zhou (2019) also assessed the intestinal microbiome of *Macaca mulatta* of different ages. They found that compared with the young group, the adult group was significantly enriched with Ruminococcaceae members, which have the function of degrading cellulose and hemicellulose, thereby providing an energy source to the host. *Lactobacillus* is significantly enriched in young individuals, and it usually acts as a probiotic to the host. Eating leaves results in higher digestion costs and lower energy returns than eating fruits, and growth, development, pregnancy, and lactation increase nutritional needs in mammals (Dufour & Sauther, 2002). Similarly, in primates, energy requirements increase with the growth and development of individuals, and young individuals have higher nutritional and energy needs than adult individuals. Similarly, Amato et al. (2014) concluded that young wild black howler monkeys have higher nutritional needs than adult individuals. This suggests that Hainan gibbons of different ages adopt different feeding strategies according to their physiological characteristics. Previous studies have confirmed that primates have a unique microbial community under conditions of no anthropogenic disturbance in the wild. However, under the influence of artificial feeding, the change in food composition changes the original microbial community; it changes along the direction similar to the composition of human microbiota, and finally converges with human intestinal microbiota (Clayton et al., 2016; Manara et al., 2019).

The imputed relative abundance of the COG and KEGG pathways revealed similarities and differences in the functional profiles of the adult and young Hainan gibbons. Compared with the intestinal microbiome of young Hainan gibbons, the intestinal microbiome of adult Hainan gibbons generally harboured a higher abundance of genes related to metabolic processes, such as metabolism of carbohydrates, amino acids, xenobiotics, inorganic ions, nucleotides, terpenoids, and polyketides. This may be due to adaptive advantages in adult Hainan gibbons, such as a stable and mature intestinal microbiome composition that allows them to utilise diverse foods efficiently. The diversity of carbohydrate active enzymes in adult and young Hainan gibbons was similar, but the abundance of carbohydrate active enzymes was different. The results showed that the abundance of GHs was the highest in the two groups, followed by GTs, which reveals that the genes related to carbohydrate transport and metabolism of intestinal microbiome in adult and young Hainan gibbons are the most abundant. This result is similar to that of other studies on animals that mainly eat leaves (Jose et al., 2017; Zhang et al., 2020). In addition, the abundance of enzymes involved in cellulose and hemicellulose degradation in adult Hainan gibbon was higher than that in young Hainan gibbon. Whereas, the abundance of enzymes involved in oligosaccharide degradation in young Hainan gibbon was higher than that in adult Hainan gibbon. This may be because the activities of cellulolytic bacteria and their secreted digestive enzymes dependent on cellulose to some extent. Therefore, the bacteria that degrade fiber, such as *Ruminococcus*, are the dominant bacteria when eating leaves containing fiber, and they secrete a large amount of cellulose- and hemicellulose-degrading enzymes. When eating more carbohydrate-rich fruits, *Prevotella*, which degrade non-fibrous plant components such as pectin and starch, are the dominant bacteria; thus, they secrete a large amount of oligosaccharide- and pectin-degrading enzymes.

## Conclusions

The Hainan gibbon is one of the most endangered primate species worldwide and the least studied gibbon species in the world. For young and adult Hainan gibbons, different feeding strategies led to differences in the composition and function of the intestinal microbiome. The adult Hainan gibbon intestinal microbiome generally harbours a high abundance of genes related to metabolic processes, such as metabolism of carbohydrates, amino acids, and nucleotides. This may be due to adaptive advantages in adult Hainan gibbons, such as a stable and mature intestinal microbiome composition that allows them to utilise diverse foods efficiently. Therefore, this study helps understand the dynamic changes in the intestinal microbiome of Hainan gibbon during youth and adulthood and plays a key role in the health monitoring and rejuvenation of its population.

## Supplemental Information

10.7717/peerj.13527/supp-1 Supplemental Information 1 Sequencing data statistics.

## Additional Information and Declarations

### Data Availability

The following information was supplied regarding data availability: The raw data are available at the NCBI Sequence Read Archive: SRR18250961.

## References

[ref-1] Amato KR, Leigh SR, Kent A, Mackie RI, Yeoman CJ, Stumpf RM, Wilson BA, Nelson KE, White BA, Garberet PA (2014). The role of gut microbes in satisfying the nutritional demands of adult and juvenile wild, black howler monkeys (*Alouatta pigra*). American Journal of Physical Anthropology.

[ref-2] Amato KR, Yeoman CJ, Kent A, Righini N, Carbonero F, Estrada A, Gaskins HR, Stumpf RM, Yildirim S, Torralba M, Gillis M, Wilson BA, Nelson KE, White BA, Leigh SR (2013). Habitat degradation impacts black howler monkey (*Alouatta pigra*) gastrointestinal microbiomes. The ISME Journal.

[ref-3] Bäckhed F, Ding H, Wang T, Hooper LV, Koh GY, Nagy A, Semenkovich CF, Gordon JI (2004). The gut microbiota as an environmental factor that regulates fat storage. Proceedings of the National Academy of Sciences of the United States of America.

[ref-4] Clayton JB, Vangay P, Huang H, Ward T, Hillmann BM, Al-Ghalith GA, Travis DA, Long HT, Tuan BV, Minh VV, Cabana F, Nadler T, Toddes B, Murphy T, Glander KE, Johnson TJ, Knights D (2016). Captivity humanizes the primate microbiome. Proceedings of the National Academy of Sciences of the United States of America.

[ref-5] Ding S, Rincon MT, Lamed R, Martin JC, McCrae SI, Aurilia V, Shoham Y, Bayer EA, Flint HJ (2001). Cellulosomal scaffoldin-like proteins from *Ruminococcu flavefaciens*. Journal of Bacteriology.

[ref-6] Dufour DL, Sauther ML (2002). Comparative and evolutionary dimensions of the energetics of human pregnancy and lactation. American Journal of Human Biology.

[ref-7] Dulski T, Kozowski K, Ciesielski S (2020). Habitat and seasonality shape the structure of tench (*Tinca tinca L*.) gut microbiome. Scientific Reports.

[ref-8] Fellowes JR, Chan BPL, Lok P, Zhou J, Chen SH, Yang SB, Ng SC (2008). Current status of the Hainan gibbon (*Nomascus hainanus*): progress of population monitoring and other priority actions. Asian Primates Journal.

[ref-9] Flint HJ (2004). Polysaccharide breakdown by anaerobic microorganisms inhabiting the mammalian gut. Advances in Applied Microbiology.

[ref-10] Geissmann T, Bleisch W (2020). Nomascus hainanus. The IUCN Red List of Threatened Species 2020. https://dx.doi.org/10.2305/IUCN.UK.2020-2.RLTS.T41643A17969392.en.

[ref-11] Han L (2019). Study on population genetic diversity of Hainan gibbon (Nomascus hainanus).

[ref-12] Hooper LV (2004). Bacterial contributions to mammalian gut development. Trends in Microbiology.

[ref-13] Hooper LV, Wong MH, Thelin A, Hansson L, Falk PG, Gordon JI (2001). Molecular analysis of commensal host-microbial relationships in the intestine. Science.

[ref-14] Jewell KA, Scott JJ, Adams SM, Suen G (2013). A phylogenetic analysis of the phylum Fibrobacteres. Systematic and Applied Microbiology.

[ref-38] Jose VL, Appoothy T, More RP, Arun AS (2017). Metagenomic insights into the rumen microbial fibrolytic enzymes in Indian crossbred cattle fed finger millet straw. AMB Express.

[ref-15] Kovacs A, Ben-Jacob N, Tayem H, Halperin E, Iraqi FA, Gophna U (2011). Genotype is a stronger determinant than sex of the mouse gut microbiota. Microbial Ecology.

[ref-16] Li YM, Hu XL, Yang S, Zhou JT, Zhang TX, Qi L, Sun XN, Fan MY, Xu SH, Cha MH, Zhang MS, Lin SB, Liu SQ, Hu DF (2017). Comparative analysis of the gut microbiota composition between captive and wild forest musk deer. Frontiers in Microbiology.

[ref-17] Li ZG, Wei FW, Zhou J (2010). Mitochondrial DNA D-loop sequence analysis and population rejuvenation of Hainan gibbons (*Nomascus hainanus*). Biodiversity Science.

[ref-18] Liu ZH, Tan CF (1990). An analysis on habitat structure of the Hainan gibbon. Acta Theriologica Sinica.

[ref-19] Manara S, Asnicar F, Beghini F, Bazzani D, Cumbo F, Zolfo M, Nigro E, Karcher N, Manghi P, Metzger MI, Pasolli E, Segata N (2019). Microbial genomes from non-human primate gut metagenomes expand the primate-associated bacterial tree of life with over 1000 novel species. Genome Biology.

[ref-20] McKenney EA, Rodrigo A, Yoder AD (2015). Patterns of gut bacterial colonization in three primate species. PLOS ONE.

[ref-21] Myer PR, Freetly HC, Wells JE, Smith TPL, Kuehn LA (2017). Analysis of the gut bacterial communities in beef cattle and their association with feed intake, growth, and efficiency. Journal of Animal Science.

[ref-22] Ochman H, Worobey M, Kuo CH, Ndjango JB, Peeters M, Hahn BH, Hugenholtz P (2010). Evolutionary relationships of wild hominids recapitulated by gut microbial communities. PLOS Biology.

[ref-23] Odamaki T, Kato K, Sugahara H, Hashikura N, Takahashi S, Xiao JZ, Abe F, Osawa R (2016). Age-related changes in gut microbiota composition from newborn to centenarian: a cross-sectional study. BMC Microbiology.

[ref-24] Owyang C, Wu GD (2014). The gut microbiome in health and disease. Gastroenterology.

[ref-25] Pafčo B, Sharma AK, Petrželková KJ, Vlčková K, Todd A, Yeoman CJ, Wilson BA, Stumpf R, White BA, Nelson KE, Leigh S, Gomez A (2019). Gut microbiome composition of wild western lowland gorillas is associated with individual age and sex factors. American Journal of Physical Anthropology.

[ref-26] Round JL, Mazmanians K (2009). The gut microbiota shapes intestinal immune responses during health and disease. Nature Reviews Immunology.

[ref-28] Sears CL (2005). A dynamic partnership: celebrating our gut flora. Anaerobe.

[ref-29] Singh RK, Chang HW, Yan D, Lee KM, Ucmak D, Wong K, Abrouk M, Farahnik B, Nakamura M, Zhu TH, Bhutani T, Liao W (2017). Influence of diet on the gut microbiome and implications for human health. Journal of Translational Medicine.

[ref-30] Sun BH, Wang X, Bernstein S, Huffman MA, Xia DP, Gu ZY, Chen R, Sheeran LK, Wagner RS, Li JH (2016). Marked variation between winter and spring gut microbiota in free-ranging Tibetan macaques (*Macaca thibetana*). Scientific Reports.

[ref-31] Tang WL, Bi Y, Jin K (2021). Composition of foraging plants of hainan gibbon in Hainan Rainforest National PaRk, China. Chinese Journal of Wildlife.

[ref-32] Tang WL, Jin K (2021). Preliminary study on night lodging habitat selection of *Nomascus hainanus* in Hainan Tropical Rainforest National Park, southern China. Journal of Beijing Forestry University.

[ref-33] Xu B, Xu WJ, Li JJ, Dai LM, Xiong CY, Tang XH, Yang YJ, Mu YL, Zhou JP, Ding JM, Wu Q, Huang ZX (2015). Metagenomic analysis of the *Rhinopithecus bieti* fecal microbiome reveals a broad diversity of bacterial and glycoside hydrolase profiles related to lignocellulose degradation. BMC Genomics.

[ref-34] Zhang ZG (2021). The Hainan Tropical Rainforest National Park-a model of precious natural resources inheritance and biodiversity conservation. Green China.

[ref-39] Zhang H, Cong LX, Wei Y, Zhao CX, Liu GW (2020). Effect of different crude fiber source diets on rumen gene function of Sika deer. Chinese Journal of Veterinary Science.

[ref-35] Zhou J (2019). Analysis of the *Macaca mulatta* gut microbes diversity in Qianlingshan Park.

[ref-36] Zhou J, Chen BL, Wei FW (2008). Responses to inter-group encounters of the Hainan Gibbon *Nomascus hainanus*. Zoological Research.

[ref-37] Zhou X, Wang B, Pan Q, Zhang J, Kumar S, Sun X, Liu Z, Pan H, Lin Y, Liu G, Zhan W, Li M, Ren B, Ma X, Ruan H, Cheng C, Wang D, Shi F, Hui Y, Tao Y, Zhang C, Zhu P, Xiang Z, Jiang W, Chang J, Wang H, Cao Z, Jiang Z, Li B, Yang G, Roos C, Garber PA, Bruford MW, Li R, Li M (2014). Whole-genome sequencing of the snub-nosed monkey provides insights into folivory and evolutionary history. Nature Genetics.

