# Supplementary material for: Comparative study on intestinal microbiome composition and function in young and adult Hainan gibbons (Nomascus hainanus)
Source: PeerJ. 2022 Jun 8;10:e13527. doi: 10.7717/peerj.13527 (PMC9188309; doi:10.7717/peerj.13527)
Supplement: Supplemental Information 1 [file peerj-10-13527-s001.docx]

**SUPPLEMENTARY MATERIALS**

Table S1. Sequencing data statistics

| Sample ID | Raw data（Mbp） | Vaild data  （Mbp） | Number of Reads | Q20 (%) | Q30 (%) | GC content（%） |
| --- | --- | --- | --- | --- | --- | --- |
| A1 | 6172.53 | 5191.79 | 17500626 | 98.00 | 94.23 | 51.15 |
| A2 | 6108.94 | 5138.78 | 17280718 | 97.90 | 93.93 | 46.85 |
| A3 | 6078.62 | 5144.27 | 17340417 | 97.89 | 93.96 | 49.89 |
| A4 | 6165.03 | 5203.75 | 17527236 | 97.91 | 93.93 | 48.06 |
| A5 | 6154.04 | 5205.25 | 17482443 | 97.85 | 93.75 | 46.14 |
| Y1 | 7082.71 | 6032.81 | 20331945 | 97.83 | 93.74 | 44.42 |
| Y2 | 6568.34 | 5563.14 | 18724500 | 97.81 | 93.73 | 45.80 |
| Y3 | 6085.85 | 5136.59 | 17260439 | 97.75 | 93.74 | 53.19 |
| Y4 | 6120.62 | 5156.13 | 17321694 | 97.76 | 93.69 | 52.37 |
| Y5 | 7117.83 | 6085.65 | 20447925 | 97.81 | 93.74 | 50.15 |
| Y6 | 6122.04 | 5216.41 | 17499946 | 97.85 | 93.75 | 46.14 |

Table S2. The top 20 relative abundance of KEGG pathways (level 2 function) for the microbial metagenome of the adult and young Hainan gibbons.

| Category | Adult (Mean±SD) | Young (Mean±SD) | P-value |
| --- | --- | --- | --- |
| Global and overview maps | 14.89%±0.20% | 14.46%±0.39% | 0.055 |
| Carbohydrate metabolism | 5.31%±0.07% | 4.70%±0.25% | 0.015 |
| Amino acid metabolism | 4.21%±0.14% | 4.06%±0.18% | 0.163 |
| Translation | 3.04%±0.15% | 3.01%±0.19% | 0.764 |
| Nucleotide metabolism | 2.93%±0.09% | 2.76%±0.08% | 0.049 |
| Membrane transport | 3.05%±0.29% | 2.09%±0.16% | 0.007 |
| Metabolism of cofactors and vitamins | 2.52%±0.18% | 2.59%±0.16% | 0.516 |
| Energy metabolism | 2.03%±0.04% | 1.95%±0.04% | 0.010 |
| Replication and repair | 1.95%±0.08% | 1.85%±0.07% | 0.051 |
| Folding, sorting and degradation | 1.21%±0.05% | 1.18%±0.07% | 0.456 |
| Glycan biosynthesis and metabolism | 1.27%±0.07 | 1.30%±0.09% | 0.241 |
| Cellular community - prokaryotes | 1.17%±0.11% | 0.92%±0.07% | 0.030 |
| Lipid metabolism | 1.03%±0.04% | 1.01%±0.06% | 0.543 |
| Metabolism of other amino acid | 1.01%±0.04% | 0.98%±0.05% | 0.356 |
| Signal transduction | 0.83%±0.05% | 0.64%±0.03% | 0.006 |
| Biosynthesis of other secondary metabolites | 0.66%±0.02% | 0.62%±0.01% | 0.020 |
| Metabolism of terpenoids and polyketides | 0.63%±0.001% | 0.60%±0.02% | 0.045 |
| Transcription | 0.25%±0.04% | 0.20%±0.04% | 0.056 |
| Xenobiotics biodegradation and etabolism | 0.16%±0.03% | 0.09%±0.03% | 0.030 |
| Cell motility | 0.13%±0.01% | 0.09%±0.01% | 0.002 |

Table S3. The top 20 relative abundance of COG categories (class function) for the microbial metagenome of the adult and young Hainan gibbons.

| Category | Adult (Mean±SD) | Young (Mean±SD) | P-value |
| --- | --- | --- | --- |
| Function unknown | 16.47%±0.36% | 16.55%±%0.53 | 0.779 |
| Replication, recombination and repair | 6.68%±0.17% | 6.58%±0.27% | 0.490 |
| General function prediction only | 6.40%±0.19% | 5.87%±0.20% | 0.001 |
| Carbohydrate transport and metabolism | 5.74%±0.24% | 5.05%±0.15% | 0.001 |
| Cell wall/membrane/envelope biogenesis | 5.62%±0.15% | 5.41%±0.08% | 0.052 |
| Amino acid transport and metabolism | 5.64%±0.13% | 5.08%±0.16% | 0.004 |
| Translation, ribosomal structure and biogenesis | 5.58%±%0.31 | 5.40%±0.36% | 0.416 |
| Transcription | 3.77%±0.23% | 3.00%±0.35% | 0.017 |
| Energy production and conversion | 3.44%±0.06% | 3.16%±0.19% | 0.012 |
| Inorganic ion transport and metabolism | 3.23%±0.06% | 3.07%±0.07% | 0.004 |
| Posttranslational modification, protein turnover, chaperones | 2.22%±0.06% | 2.35%±0.09% | 0.022 |
| Nucleotide transport and metabolism | 2.25%±0.06% | 2.22%±0.06% | 0.312 |
| Defense mechanisms | 2.22%±0.15% | 1.87%±0.12% | 0.002 |
| Signal transduction mechanisms | 2.01%±0.09% | 1.77%±0.07% | 0.004 |
| Coenzyme transport and metabolism | 1.77%±0.13% | 1.83%±0.09% | 0.411 |
| Lipid transport and metabolism | 1.42%±0.03% | 1.43%±0.03% | 0.774 |
| Intracellular trafficking, secretion, and vesicular transport | 0.99%±0.02% | 0.94%±0.13% | 0.464 |
| Cell cycle control, cell division, chromosome partitioning | 0.85%±0.04% | 0.82%±0.11% | 0.507 |
| Secondary metabolites biosynthesis, transport and catabolism | 0.47%±0.02% | 0.46%±0.04% | 0.659 |
| Cell motility | 0.20%±0.02% | 0.20%±0.03% | 0.968 |
